# Technology-supported behavior change interventions for reducing sodium intake in adults: a systematic review and meta-analysis

**DOI:** 10.1038/s41746-024-01067-y

**Published:** 2024-03-18

**Authors:** Yong Yang Yan, Lily Man Lee Chan, Man Ping Wang, Jojo Yan Yan Kwok, Craig S. Anderson, Jung Jae Lee

**Affiliations:** 1 School of Nursing, Li Ka Shing Faculty of Medicine, The University of Hong Kong, Hong Kong, China; 2 The George Institute for Global Health, Faculty of Medicine, University of New South Wales, Sydney, Australia

**Keywords:** Hypertension, Nutrition, Lifestyle modification, Risk factors

## Abstract

The effects of technology-supported behavior change interventions for reducing sodium intake on health outcomes in adults are inconclusive. Effective intervention characteristics associated with sodium reduction have yet to be identified. A systematic review and meta-analysis were conducted, searching randomized controlled trials (RCTs) published between January 2000 and April 2023 across 5 databases (PROSPERO: CRD42022357905). Meta-analyses using random-effects models were performed on 24-h urinary sodium (24HUNa), systolic blood pressure (SBP), and diastolic blood pressure (DBP). Subgroup analysis and meta-regression of 24HUNa were performed to identify effective intervention characteristics. Eighteen RCTs involving 3505 participants (51.5% female, mean age 51.6 years) were included. Technology-supported behavior change interventions for reducing sodium intake significantly reduced 24HUNa (mean difference [MD] −0.39 gm/24 h, 95% confidence interval [CI] −0.50 to −0.27; *I*^2^ = 24%), SBP (MD −2.67 mmHg, 95% CI −4.06 to −1.29; *I*^2^ = 40%), and DBP (MD −1.39 mmHg, 95% CI −2.31 to −0.48; *I*^2^ = 31%), compared to control conditions. Interventions delivered more frequently (≤weekly) were associated with a significantly larger effect size in 24HUNa reduction compared to less frequent interventions (>weekly). Other intervention characteristics, such as intervention delivery via instant messaging and participant-family dyad involvement, were associated with larger, albeit non-significant, effect sizes in 24HUNa reduction when compared to other subgroups. Technology-supported behavior change interventions aimed at reducing sodium intake were effective in reducing 24HUNa, SBP, and DBP at post-intervention. Effective intervention characteristics identified in this review should be considered to develop sodium intake reduction interventions and tested in future trials, particularly for its long-term effects.

## Introduction

Excessive sodium intake (≥2 gm of sodium or equivalent to ≥5 gm of salt intake daily^1^) is the leading dietary risk factor of hypertension (HTN) and cardiovascular disease (CVD)^1,2^, causing 1.65 million global deaths related to CVD annually^2^. The World Health Organization (WHO) recommends adults consume less than 2 gm of sodium daily^3^, as reducing sodium intake can lower blood pressure (BP)^4,5^, thereby reducing death and disability attributable to CVD^6^. However, most adults consume an average of 3.5–5.5 gm sodium daily^3^, which is significantly higher than the recommended amount.

Changing consumers’ sodium intake behavior through education is a sodium reduction strategy proposed by the WHO^7^. A narrative systematic review reported that behavior change interventions improved sodium intake behavior and/or reducedsodium intake^8^. A meta-analysis further confirmed the effect of sodium reduction behavior change interventions^9^. However, these two reviews primarily included interventions with a face-to-face delivery mode. Technology-supported interventions, targeting to modify individuals’ sodium intake behavior by using technological tools (e.g., telephone, video, web/mobile applications, and digital devices)^9,10^, have been increasingly used to reduce sodium intake in order to reach a wider population and reduce costs^7,9^. A narrative systematic review identified that 64% of interventions supported by mobile applications (app) or short message services (SMS) have beneficial effects on sodium reduction^11^. However, no meta-analysis has been conducted to quantify the intervention effects on sodium intake and BP^11^. Given increasing studies of technology-supported interventions in sodium intake reduction have been published in recent years^12,13^, it is timely to conduct a systematic review and meta-analysis to synthesize the evidence.

Identifying effective intervention characteristics, such as delivery technology type, delivery frequency, and behavior change techniques (BCTs), can help develop more effective behavior change interventions^14^ to address the complexity^15^ and resource consumption^8^ involved in changing an individual’s sodium intake behavior. BCTs are the smallest replicable intervention components intended to alter or redirect causal processes that regulate behavior^16,17^. Specifying the BCTs applied in behavior change interventions can facilitate a better understanding of the mechanisms of initiating and sustaining desired behaviors and the replication of interventions across diverse settings^16,17^. Yet, the effective intervention characteristics associated with sodium reduction, including BCTs, have not been explored.

This systematic review and meta-analysis aimed to evaluate the effects of technology-supported behavior change interventions for reducing sodium intake on sodium reduction, systolic blood pressure (SBP), and diastolic blood pressure (DBP) and explore effective intervention characteristics associated with sodium reduction.

## Results

### Study characteristics

**Fig. 1:**
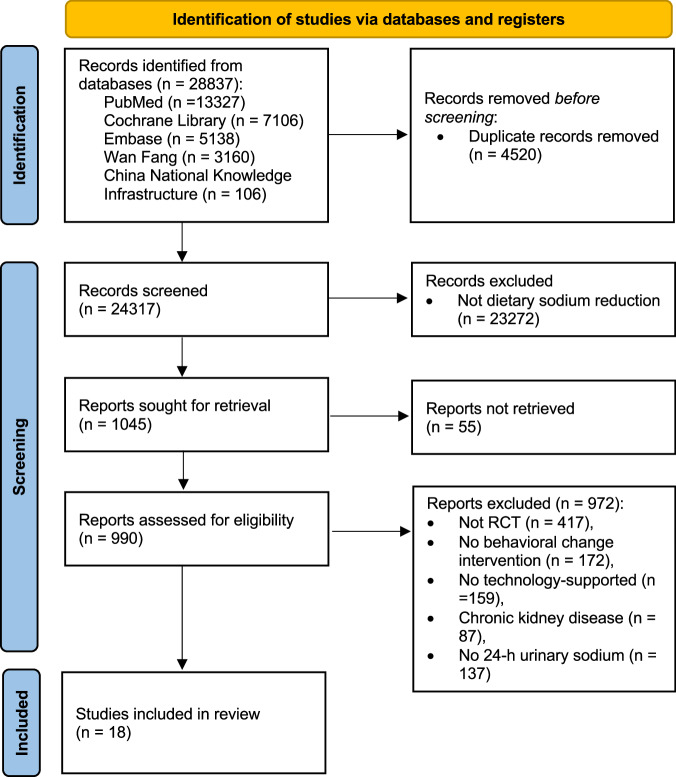
PRISMA flow chart of study selection. The flow chart, following the Preferred Reporting Items for Systematic Reviews and Meta-Analyses (PRISMA) guidelines, illustrates the detailed process of study search and selection.

A total of 28,837 articles were retrieved, and 18 randomized controlled trials (RCTs) (19 comparisons) were included in the final review (Fig. 1)^12,13,18–33^. Study characteristics are shown in Table 1 and Supplementary Table 1. The RCTs were published between 2005 and 2023 from 10 countries. Nine RCTs (3 each) were conducted in Japan^25–27^, Thailand^28,30,31^, and the United States^19,20,23^, with over half (*n* = 10) conducted in Asia^12,22,24–28,30–32^ (Supplementary Fig. 1). The review included 3505 participants, with 51.5% (*n* = 1805) being female (sex not disclosed in one RCT^28^). Participants’ mean age ranged from 28.5 to 66.3 years (median 54.5; mean 51.6 [standard deviation, SD 12.2]). Nine RCTs recruited hypertensive patients^13,18,19,22,25,28,30,32,33^, three recruited healthy/normative participants^23,24,29^, two recruited patients with heart disease (i.e., heart failure^20^, prior acute coronary syndrome, revascularization, or exertion angina^21^), and four recruited hypertensive and healthy/normative participants^12,26,27,31^. The intervention settings were nonhealthcare settings (*n* = 12)^12,18,19,21–26,28,29,33^, healthcare settings (*n* = 3)^20,30,31^, and the combination of nonhealthcare and healthcare settings (*n* = 3)^13,27,32^. Nonhealthcare settings included homes, grocery stores, restaurants, schools, and worksites. The intervention duration ranged from 1 to 12 months (median 2; mean 2.8 [SD 2.6]), and the follow-up duration ranged from 0 to 9 months (median 0; mean 0.8 [SD 2.1]).

**Table 1:** Study Characteristics

Author, Year, Country	Sample Size (IG, CG), No. Female (%)	Mean Age (years)	Health Status	Intervention Setting	Intervention + Follow-up Duration	Technology Type
Cornelio^18^, 2014, Brazil	119 (62, 57), 119 (100.00%)	59.00	HTN	Nonhealthcare setting	2 months + 1 month	Telephone call
Dorsch^19^, 2020, USA	50 (24, 26), 30 (60.00%)	57.43	HTN	Nonhealthcare setting: home, grocery store, and restaurant	2 months + 0 month	App developed for sodium reduction (LowSalt4Life)
Dunbar^20^, 2005, USA	61 (32, 29), 28 (45.90%)	61.00	Heart disease	Healthcare setting	3 months + 0 month	Telephone call + Video (Primary type: Telephone call)
Eyles a^21^, 2017, New Zealand	66 (33, 33), 11 (16.67%)	64.00	Heart disease	Nonhealthcare setting: grocery store	1 month + 0.5 month	App developed for sodium reduction (SaltSwitch) + Email + Telephone call (Primary type: SaltSwitch)
Eyles b^33^, 2023, New Zealand	168 (84, 84), 74 (49.01%)	54.50	HTN	Nonhealthcare setting: home and grocery store	3 months + 0 month	App developed for sodium reduction (SaltSwitch) + SMS + Video (Primary type: SaltSwitch)
He^12^, 2022, China	1184 (594, 590), 633 (53.46%)	45.80	HTN + Healthy adults	Nonhealthcare setting: home and school	12 months + 0 month	App developed for sodium reduction (AppSalt) + Instant messaging App (WeChat) (Primary type: AppSalt)
Hwang^22^, 2014, South Korea	245 (119, 126), 123 (50.20%)	49.56	HTN	Nonhealthcare setting	2 months + 2 months	Telephone call
Ipjian^23^, 2017, USA	30 (15, 15), 23 (76.67%)	34.40	Healthy population	Nonhealthcare setting	1 month + 0 month	App developed for sodium reduction (MyFitnessPal)
Jarrar^24^, 2022, United Arab Emirates	121 (IG1: 41, IG2: 41, CG: 39), 60 (49.59%)	28.50	Healthy population	Nonhealthcare setting	1.5 months + 1 month	IG1: Instant messaging App (WhatsApp), IG2: Email
Morikawa^25^, 2011, Japan	41 (22, 19), 0 (0.00%)	47.74	HTN	Nonhealthcare setting: worksite	1 month + 0 month	Digital self-monitoring sodium device + Email (Primary type: the device)
Nakadate^26^, 2018, Japan	50 (28, 22), 35 (70.00%)	53.54	HTN + Healthy adults	Nonhealthcare setting: home	3 months + 0 month	Digital self-monitoring sodium device
Riches^13^, 2021, UK	47 (31, 16), 30 (63.83%)	65.00	HTN	Nonhealthcare + healthcare setting	1.5 months + 0.5 month	App developed for sodium reduction (SaltSwap) + SMS (Primary type: SaltSwap)
Takada^27^, 2018, Japan	158 (79, 79), 105 (66.46%)	62.45	HTN + Healthy adults	Nonhealthcare + healthcare setting	1 month + 0 month	Digital self-monitoring sodium device
Thatthong^28^, 2020, Thailand	67 (32, 35), NA	40.94	HTN	Nonhealthcare setting: worksite	2 months + 0.5 month	Instant messaging App (LINE)
Toft^29^, 2020, Denmark	112 (63, 24), 46 (52.87%)	40.68	Healthy population	Nonhealthcare setting	4 months + 0 month	Telephone call + Email (Primary type: telephone call)
Wiriyatanakorn^30^, 2021, Thailand	90 (45, 45), 49 (54.44%)	62.90	HTN	Healthcare setting	2 months + 0 month	Digital self-monitoring sodium device
Yokokawa^31^, 2020, Thailand	753 (374, 379), 367 (48.74%)	66.25	HTN + Healthy population	Healthcare setting	3 months + 9 months	Digital self-monitoring sodium device
Yuan^32^, 2019, China	168 (84, 84), 72 (42.86%)	57.50	HTN	Nonhealthcare + healthcare setting	6 months + 0 month	Instant messaging App (WeChat) + Telephone call + SMS (Primary type: WeChat)

*App* application, *CG* control group, *HTN* hypertension, *IG* intervention group, *NA* not available, *No.* number, *SMS* short message service, *UK* the United Kingdom, *USA* the United States of America.

Note: Primary technology type was defined as the most frequently used or the predominant technology used in the intervention.

Seven types of technological tools were identified: mobile apps developed for sodium reduction (*n* = 6)^12,13,19,21,23,24,33^, telephone calls (*n* = 6)^18,20–22,29,32^, digital self-monitoring sodium devices (*n* = 5)^25–27,30,31^, instant messaging apps (*n* = 4)^12,24,28,32^, emails (*n* = 4)^21,24,25,29^, SMS (*n* = 3)^13,32,33^, and videos (*n* = 2)^20,33^. Eight RCTs used partially technology-supported interventions with face-to-face components^12,13,18,20,29–32^. Nine RCTs used an individual-based delivery mode^13,19,22–25,28,32,33^, followed by group-based mode (*n* = 4)^12,18,30,31^, dyad-based mode (*n* = 3, all were participant-family dyads)^20,27,29^, and mixed mode (*n* = 2)^21,26^. Six RCTs involved participants’ family members^12,20,21,26,27,29^. Different intervention frequencies were found, including ≤weekly (*n* = 7)^22–24,26,28,32,33^, using mobile apps developed for sodium reduction when food shopping (*n* = 4)^13,19,21,33^, >weekly (*n* = 3)^12,18,24^, and four not identified^20,27,29,31^. Dietitians were the most common professionals delivering interventions (*n* = 5)^20,22,27,30,31^, followed by nurses (*n* = 4)^13,18,20,32^, nutritionists (*n* = 2)^18,29^, physicians (*n* = 1)^27^, healthcare assistants (*n* = 1)^13^, research assistants (*n* = 1)^28^, and trained teachers (*n* = 1)^12^. Only six RCTs employed theoretical frameworks^13,18–20,27,29^. Three self-estimation methods for sodium intake were identified: mobile apps developed for sodium reduction (*n* = 6)^12,13,19,21,23,33^, self-monitoring sodium devices (*n* = 5)^25–27,30,31^, and salt-restriction spoons/scales (*n* = 2)^18,32^. Ten studies included an inactive control group (e.g., general health education without focusing on sodium reduction or no treatment)^12,18,19,21,24,25,28,29,31,32^, while eight studies had an active control group (e.g., salt reduction advice leaflet)^13,20,22,23,26,27,30,33^. Intervention groups had a sodium reduction ranging from −2.77 gm/24 h to +0.32 gm/24 h, indicating the sodium reduction percentages from −46.94% to +8.85% (median −14.95%; mean −14. 56% [SD 11.99]).

Regarding BCT identification, 35 BCTs from 13 groupings were identified in the intervention groups from 18 included RCTs (Supplementary Table 2.1 and Table 2.2). BCTs identified per RCT ranged from 5 to 24 (median 10.00; mean 11.68 [SD 4.84]). The five most frequently identified BCTs were 4.1 ‘instruction on how to perform the behavior’ (*n* = 18), 6.1 ‘demonstration of the behavior’ (*n* = 17), 8.1 ‘behavioral practice/rehearsal’ (*n* = 17), 8.3 ‘habit formation’ (*n* = 15), 1.1 ‘goal setting(behavior)’ (*n* = 12), 1.4 ‘action planning’ (*n* = 12), 2.2 ‘feedback on behavior’ (*n* = 10), and 5.1 ‘information about health consequences’ (*n* = 10) (Supplementary Table 2.3).

### Risk of bias assessment

Overall quality judgments rated 33% of RCTs with a low risk of bias (*n* = 6)^12,13,21,29,30,33^, 50% with some concerns of bias (*n* = 9)^19,20,22–27,31^, and 17% with a high risk of bias (*n* = 3) (Supplementary Fig. 2)^18,28,32^. A high risk of bias was identified in 6 RCTs in Domain 2 (deviation from intended interventions) and 1 RCT in Domain 3 (missing outcome data).

### Meta-analysis of the 24HUNa, SBP, and DBP

**Fig. 2:**
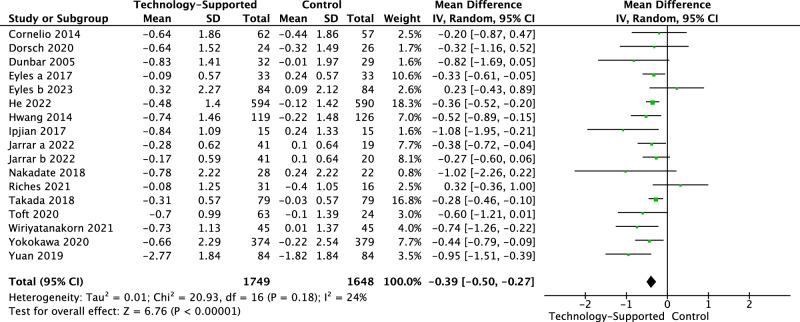
Forest plot of 24-h urinary sodium. The unit of 24-h urinary sodium (24HUNa) was ‘gm/24 h’. The ‘Mean’ (change score or change from baseline) of 24HUNa within each group was calculated by subtracting the baseline mean from the post-intervention mean. The ‘Mean Difference’ refers to the comparison of mean values between two groups. Post-intervention data was used in the meta-analysis as the majority of studies did not assess outcomes during follow-up periods.

**Fig. 3:**
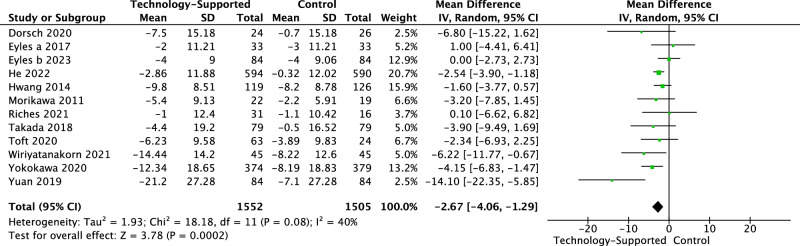
Forest plot of systolic blood pressure. The unit of systolic blood pressure (SBP): mmHg. The ‘Mean’ (change score or change from baseline) of SBP within each group was calculated by subtracting the baseline mean from the post-intervention mean. The ‘Mean Difference’ refers to the comparison of mean values between two groups. Post-intervention data was used in the meta-analysis as the majority of studies did not assess outcomes during follow-up periods.

**Fig. 4:**
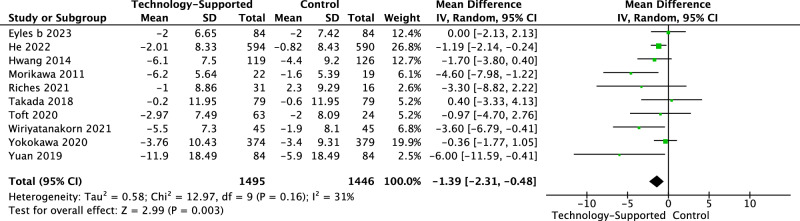
Forest plot of diastolic blood pressure. The unit of diastolic blood pressure (DBP): mmHg. The ‘Mean’ (change score or change from baseline) of DBP within each group was calculated by subtracting the baseline mean from the post-intervention mean. The ‘Mean Difference’ refers to the comparison of mean values between two groups. Post-intervention data was used in the meta-analysis as the majority of studies did not assess outcomes during follow-up periods.

Technology-supported behavior change interventions significantly reduced 24HUNa (MD −0.39 gm/24 h, 95% CI −0.50 to −0.27; *I*^2^ = 24%; Fig. 2), at a rate equivalent to a salt reduction of 0.98 gm/24 h(95% CI −1.25 to −0.68) compared to active or inactive control conditions^12,13,18–24,26,27,29–33^. The effect size of 24HUNa was *g* −0.32 (95% CI to −0.42 to −0.21), representing a small to medium effect based on the absolute value of *g*. No publication bias was detected by the Begg test (*p* = 0.13) and the Egger test (*p* = 0.18). SBP significantly decreased following the intervention (MD −2.67 mmHg, 95% CI −4.06 to −1.29; *I*^2^ = 40%; Fig. 3)^12,13,19,21,22,25,27,29–33^, with a small to medium effect based on the absolute value of *g* (*g* −0.22, 95% CI −0.29 to −0.15). The Begg test (*p* = 0.20) and the Egger test (*p* = 0.13) did not discover publication bias. A significant decrease was also found in DBP(MD −1.39 mmHg, 95%CI −2.31 to −0.48; *I*^2^ = 31%; Fig. 4)^12,13,22,25,27,29–33^, with a small effect based on the absolute value of *g* (*g* −0.15, 95% CI −0.25 to −0.0). Neither the Begg test (*p* = 0.22) nor the Egger test (*p* = 0.08) indicated publication bias.

Overall, sensitivity analyses supported the robustness of the effects on 24HUNa, SBP, and DBP. The intervention effects were not significantly changed after excluding RCTs with high risk of bias regarding 24HUNa (MD −0.36 gm/24 h, 95% CI −0.47 to −0.26 and *g* −0.32, 95% CI −0.43 to −0.20), SBP (MD −2.33 mmHg, 95% CI −3.29 to −1.38 and *g* −0.20, 95% CI −0.28 to −0.13), and DBP (MD −1.23 mmHg, 95% CI −2.05 to −0.40 and *g* −0.13, 95% CI −0.24 to −0.03).

### Subgroup analysis and meta-regression of the 24HUNa

The results of subgroup analyses are reported in Supplementary Table 3. Intervention frequency significantly affected the effects on 24HUNa (*p* = 0.03), with more frequent interventions (≤weekly) achieving a larger effect than less frequent interventions (>weekly). Additionally, applying BCT 6.1 ‘demonstration of the behavior’ (*p* = 0.04) and 8.1 ‘behavioral practice/rehearsal’ (*p* = 0.04) resulted in significantly greater reductions in 24HUNa, compared to not using these techniques. No significant differences were detected in other subgroups of intervention characteristics. However, relatively but not significantly larger effect sizes of 24HUNa were shown in the following subgroups: normative participants (*g* −0.57), use of instant messaging apps (*g* −0.53), multidisciplinary professionals for intervention delivery (*g* −0.35), dyad-based intervention delivery mode (*g* −0.50), family member involvement (*g* −0.33), entirely technology-supported interventions (*g* −0.37), healthcare setting (*g* −0.34), using digital self-monitoring sodium devices as the method of self-estimating sodium intake (*g* −0.37), individually applying the BCTs of 1.1 ‘goal setting(behavior)’ (*g* −0.32), 2.3 ‘self-monitoring of behavior’ (*g* −0.34), 2.6 ‘biofeedback’ (*g* −0.37), 3.2 ‘social support (practical)’ (*g* −0.35), 8.3 ‘habit formation’ (*g* −0.36), and 9.1 ‘credible source’ (*g* −0.33).

Meta-regressions suggested that sample size, proportion of female participants, mean age, intervention duration, follow-up duration, and number of BCTs identified in each RCT were not associated with 24HUNa reduction (Supplementary Table 4).

## Discussion

This is the first meta-analysis to evaluate the effects of technology-supported behavior change interventions for reducing sodium intake on 24HUNa, SBP, and DBP and identify the BCTs used in behavior change interventions for sodium intake reduction. Subgroup analyses showed that intervention frequency and the BCTs of 6.1 ‘demonstration of the behavior’ and 8.1 ‘behavioral practice/rehearsal’ were significantly associated with the effect on 24HUNa. Other effective intervention characteristics in reducing 24HUNa, such as primary technology type and intervention delivery mode, were also identified.

Significant 24HUNa reduction was observed (MD −0.39 gm/24 h) in this meta-analysis. The result is comparable to a finding from a meta-analysis (MD −0.46 gm/24 h), in which included interventions were primarily delivered in a face-to-face format^9^. This suggests that technology-supported behavior change interventions can serve as an effective alternative to traditional face-to-face interventions. Reducing sodium intake by 0.39 gm/24 h by behavior change has considerable public health implications. It was estimated that a 0.4 gm/24 h sodium reduction would substantially reduce 9 million CVD events and save 4 million lives in the Chinese population by 2030^34^. The significant reductions of 2.67 mmHg in SBP and 1.39 mmHg in DBP identified in this meta-analysis are comparable to the findings of a recent meta-analysis, which found that health education interventions significantly reduced SBP and DBP by 2.8 and 2.1 mmHg, respectively^4^. Similarly, another meta-analysis found sodium reduction via dietary modifications reduced 2.9 mmHg in SBP and 1.2 mmHg in DBP^35^. The similar magnitudes of BP reduction in this study suggest that technology-supported interventions are as efficacious as face-to-face-delivered interventions in controlling BP. These effects on 24HUNa, SBP, and DBP are robust, as confirmed by the leave-one-out sensitivity analysis and excluding RCTs with a high risk of bias. It is important to note that the effects on 24HUNa and BP reduction in this meta-analysis represent post-intervention effects. These effects were assessed over relatively short intervention durations (mean 2.8 months and median 2 months), without assessment of longer-term follow-up effects. Future RCTs are needed to evaluate and report the long-term effects on 24HUNa and BP. Although technology-supported interventions can supplement current sodium reduction efforts, the average proportion of sodium reduction (14.56%) in the intervention group found in this review would not be sufficient to reach the WHO’s goal of a 30% reduction in the population’s sodium intake by 2030. Future research should explore the integration of technology-supported interventions into the sodium reduction initiatives recommended by the WHO, such as food reformulation and front‑of‑pack nutrition labeling initiatives^7,11^. By incorporating these technological advancements, it is possible to amplify the effects of current multi-faceted sodium reduction initiatives, ultimately resulting in more significant synergistic health benefits. For instance, leveraging technology such as widely used instant messaging apps (e.g., WhatsApp and WeChat) to distribute educational content on sodium reduction could enhance awareness among the broader population and foster greater acceptance of low-sodium products resulting from food reformulation^7^. This approach can also improve their comprehension and use of front-of-pack nutrition labels, empowering them to make informed choices about low-sodium foods^7,11^. Given the widespread accessibility of technology such as smartphones, this approach can be readily applied to patients in healthcare settings as well as to community-dwelling individuals in non-healthcare environments. Consequently, the technology-supported strategy not only bolsters individual efforts to reduce sodium intake but also reinforces the effectiveness of the food industry and governmental efforts in such food reformulation and nutrition labeling initiatives.

Effective intervention characteristics identified in this review provide evidence for guiding the development of future interventions. Significant 24HUNa reductions were found among hypertensive, normative participants, and a combination of both, suggesting the feasibility of using technology-supported interventions for hypertensive patients and the general population. Regardless of the delivery mode, whether as individuals, dyads, or groups—all modes were effective in reducing 24HUNa. Dyad-based interventions (i.e., participant-family dyads) were likely to have a greater effect size (*g* −0.50) than individual-based (*g* −0.30) and group-based (*g* −0.24) interventions. This result is consistent with another subgroup analysis in this study, which found that involving family members in participants’ sodium reduction had a relatively larger effect size (*g* −0.33) compared to interventions in which family members did not participate (*g* −0.28). One possible reason is that individual sodium intake is affected by social environment (e.g., a family’s eating habits^36^). Involving family members to support individual sodium reduction, such as changing the family’s eating habits or the home chef’s cooking behavior, might be a promising strategy to reduce sodium intake^20,37^. Technology-supported interventions were also effective in reducing 24HUNa in healthcare (*g* −0.34) and non-healthcare settings (*g* −0.30), irrespective of the presence of face-to-face intervention components, suggesting the potential for technology-supported interventions to be used as a standalone approach for reducing sodium intake. Compared to partially technology-supported interventions (*g* −0.29), entirely technology-supported interventions were found to produce a stronger effect (*g* −0.37) and may be less resource-consuming. Thus, implementing technology-supported interventions in different sub-populations and settings can be an effective public health approach in primary prevention and secondary management of excessive sodium intake.

Effective reductions in 24HUNa were observed regarding different technology types, including instant messaging apps (*g* −0.53), telephone calls (*g* −0.33), apps developed for sodium reduction (*g* −0.21), and digital self-monitoring sodium devices (*g* −0.37). Among these types, interventions delivered via instant messaging apps, such as WhatsApp and WeChat, had a relatively larger effect size on sodium reduction. Instant message-delivered interventions, which can be delivered through various formats (e.g., text, image, or voice) and cater to the participants’ preferred time and frequency, provide participants with more personalized and real-time feedback from health professionals, leading to improved outcomes^38,39^. Notably, interventions delivered weekly or less than weekly had a significantly larger effect size (*g* −0.48, more frequent) over more than weekly (*g* −0.25, less frequent) and are recommended for future trials. As sodium intake is highly associated with daily diet consumption^1,40^, interventions that are delivered more frequently may serve as a constant reminder for individuals to adopt healthier sodium intake behaviors in their daily lives, potentially leading to better sodium reduction outcomes. Except for more frequent delivery, innovative methods of self-estimation of sodium intake, such as apps developed for sodium reduction (*g* −0.21) and digital self-monitoring sodium devices (*g* −0.37) can also be applied in the intervention. As approximately 55% of the population is unaware of their daily sodium intake and tends to underestimate it^41,42^, providing individuals with more accurate feedback on their sodium intake can help them understand their sodium intake conditions and make informed decisions to initiate, adjust, or maintain their sodium reduction behavior^15^. Dietitians, nurses, nutritionists, and physicians can independently practice (*g* −0.31) or collaborate in a multidisciplinary team (*g* −0.35) to reduce individuals’ sodium intake, which later potentially results in a greater effect size on sodium reduction. A meta-analysis also highlighted the importance of involving healthcare professionals in achieving better outcomes in sodium reduction interventions^8^.

Compared to without BCT 6.1 ‘demonstration of the behavior’ (*g* 0.27), using BCT 6.1 (*g* −0.33) was significantly associated with larger reductions in 24HUNa, where providing observable samples, such as a cookbook of low-sodium recipes^18^ or photographs of high-sodium food^29^, facilitated participants to imitate the desired behavior by increasing their knowledge and skills to reduce sodium intake^17^. BCT 8.1 ‘behavioral practice/rehearsal’ (*g* −0.33) was also significantly associated with 24HUNa reduction compared to without BCT 8.1 (*g* 0.27), where participants practiced the desired behavior to improve their self-efficacy on sodium reduction^17,43^. The results should be interpreted with caution, considering that the two significantly effective BCTs were identified across 15 studies, compared to only one study that did not employ these techniques. Only one original RCT clearly stated the BCTs used^13^, and only 35 out of the 93 BCTs were identified, implying that BCTs are not widely explored in behavior change interventions for sodium reduction. Future trials aimed at changing behavior for sodium reduction may employ effective BCTs identified in this study and explore other BCTs.

This review adhered to PRISMA guidelines with a rigorous analysis of the overall effects, including publication bias examination, sensitivity analysis, and excluding RCT with high risks of bias. This review included only RCTs where the sodium intake was evaluated by 24HUNa, which provides an objective appraisal of the evidence on sodium reduction. The focus was exclusively on behavior modifications related to sodium intake, without examining interventions that included other HTN-related lifestyle modifications. This approach minimized the potential influence of confounding variables or moderators on BP reduction and provided a more targeted assessment of the effect of sodium intake behavior change on BP reduction.

This study has several limitations. The participant numbers in some subgroups were not evenly distributed. The results of subgroup analyses should be interpreted with caution as statistical tests may not detect significant differences between subgroups, or the results may be biased toward the larger subgroup. Insufficient intervention description in some original RCTs, especially on BCTs, limited subsequent analyses of the intervention characteristics associated with 24HUNa.

In conclusion, our systematic review and meta-analysis showed that technology-supported behavior change interventions for reducing sodium intake are effective in reducing 24HUNa, SBP, and DBP at post-intervention in adults. Further trials need to test the effective characteristics, such as intervention frequency (≤weekly), technology (instant messaging apps), delivery mode (participant-family dyad), and long-term effects on sodium and BP reduction.

## Methods

The systematic review and meta-analysis followed the Preferred Reporting Items for Systematic Reviews and Meta-Analyses guidelines^44^ (PRISMA, Supplementary Table 5) and was registered on the International Prospective Register of Systematic Reviews (PROSPERO registration number: CRD42022357905).

### Search strategy

Both English-language (PubMed, Cochrane Library, and Embase) and Chinese-language databases (Wan Fang and China National Knowledge Infrastructure) were searched for relevant articles written in English or Chinese published between 1 January 2000 and 13 April 2023. Medical subject headings(MeSH) and keywords related to ‘technology’, ‘sodium’, ‘salt’, and ‘reduction’ were used for the literature search. A manual search of reference lists was also conducted (Supplementary Table 6).

### Study selection

The eligibility criteria based on population, intervention, comparison, outcomes, and study (PICOS) were applied. Population: adults (≥18 years). Participants with chronic kidney disease were excluded as the WHO’s sodium intake recommendation is not applicable to all these patients^45^. Intervention: technology-supported interventions targeting to change individuals’ sodium intake behavior by partially (i.e., combined with face-to-face intervention components) or entirely utilizing technological tools, such as telephone, video, web/mobile apps, and digital devices. Interventions that addressed modifications to both sodium intake behavior and other HTN risk factors (e.g., physical inactivity) were excluded. Comparison: participants in the control groups received either active control^46^ (e.g., sodium reduction education conducted contemporaneously with the intervention group) or inactive control (e.g., usual/standard treatment or no treatment). Outcomes: the original RCTs must report 24-h urinary sodium (24HUNa), which is the gold standard for estimating daily sodium intake^47^, regardless of whether it was estimated from 24-h urine or spot urine samples and reported together with BP readings. Study: RCTs, excluding review, abstract, and protocol papers.

### Data extraction

The RCTs were independently assessed by two reviewers (Y.Y.Y. and C.M.L.) for eligibility, information extraction, and quality assessment. Disagreements during these processes were resolved by a third reviewer (L.J.J.).

The extracted information: author, year, country, sample size, number and proportion of female participants, mean age, participants’ health status, intervention setting, intervention duration, follow-up duration, technology type, primary technology type (defined as the most frequently used or the predominant technology used in the intervention), urine sample type, intervention delivery mode (defined as individual-based, dyad-based, or group-based interventions, which interventions were delivered to 1, 2, or ≥3 participants, respectively^48^), number of family members involved, intervention frequency, intervention delivery professional, partially technology-supported or entirely technology-supported, method of self-estimation of sodium intake, and proportion of sodium reduction in the intervention groups (calculated by: 100%*[24HUNa at post-intervention − 24HUNa at baseline]/24HUNa at baseline^9^).

The BCTs used in the original RCTs were identified by Michie’s BCT Taxonomy (BCTTv1)^16^, which contains 93 BCTs with unique codes and definitions. Intervention contents in the intervention groups were mapped to BCTTv1. The coding of each BCT was based on whether it was explicitly described in the intervention and assigned a ‘+’ if the content description met the corresponding definition. The BCTs were first identified by one reviewer (Y.Y.Y.), then double-checked and confirmed by another reviewer (C.M.L.).

All units of 24HUNa were converted to ‘gm/24 h for meta-analysis^47^. Post-intervention data was used in the meta-analysis as the majority of RCTs did not assess outcomes during follow-up periods. The change score was calculated by subtracting the baseline mean from the post-intervention mean within each group^49^ and used in the meta-analysis. Corresponding SDs were calculated from the sample size, standard errors, confidence intervals, or *t*, *z*, or *p* values^49^. If the SD could not be computed from the aforementioned values, the correlation coefficients were computed instead^49^. Due to the unavailability of similar meta-analyses to refer to in calculating coefficients, data from a single RCT that aimed to reduce participants’ sodium intake by changing their sodium intake behavior through a mobile app were used to compute the correlation coefficients, as this RCT was reported in considerable detail, including change scores and SD needed to compute the correlation coefficients^12^. The correlation coefficients used to calculate SD of 24HUNa, SBP, and DBP were 0.5, 0.8, and 0.7, respectively. Sensitivity analyses were performed using different correlation coefficients to examine the robustness of the overall estimates^49^.

### Risk of bias assessment

The quality of included RCTs was assessed by the Cochrane risk-of-bias tool for randomized trials (ROB2)^50^, which categorizes risk of bias as low, some concerns, or high.

### Meta-analysis

Random-effect models with the inverse variance method were applied to pool estimated effects on 24HUNa (primary outcome), SBP, and DBP. The estimates were reported as mean difference (MD), 95% confidence interval (CI), and *I*^2^ statistics. Standardized mean difference (SMD), estimated using adjusted Hedges’*g*, was used to determine effect size magnitude^51^. In this review, a negative *g* indicates the intervention group mean is lower than the control mean^52^, with the absolute value of *g* = 0.2, 0.5, and 0.8 for small, medium, and large effects, respectively^53^. Publication bias was evaluated by the Egger sand Begg tests^54^, and if identified, the estimated effects were adjusted by the trim and fill method^55^. Sensitivity analysis was performed by the leave-one-out method to verify the robustness of the estimated effects^49^. Review Manager (version 5.4) and R Studio (‘metafor’ package, version 2022.07.1) were used for the meta-analysis. A *p*-value of <0.05 (two-sided) was considered statistically significant.

### Subgroup analysis and meta-regression

The subgroup analyses were conducted: BP of participants (hypertensive vs. normative vs. hypertensive+normative)^4,9^, primary technology type (instant messaging app vs. telephone call vs. app developed for sodium reduction vs. digital self-monitoring sodium device)^4,9,11^, intervention delivery professional (multidisciplinary vs. unidisciplinary), intervention delivery mode (individual- vs. dyad- vs. group-based)^48^, family members involved (yes vs. no)^56^, partially or entirely technology-supported (partially vs. entirely)^57^, intervention frequency (≤weekly vs. >weekly)^4,11^, intervention settings (nonhealthcare vs. healthcare vs. nonhealthcare+healthcare), method of self-estimation of sodium intake (app developed for sodium reduction vs. digital self-monitoring sodium device vs. salt-restriction spoon/scale), urine sample used for estimating sodium intake (24-h urine vs. spot urine), control group (active control vs. inactive control)^46^, and whether the BCT was used (yes vs. no)^14^. The BCTs identified in at least five original RCTs were eligible for subgroup analyses to avoid inflation of the results from BCTs that were only sporadically used in the original RCTs^14^. Meta-regression was performed on continuous variables, including sample size, the proportion of female participants, mean age^58^, intervention duration^58^, follow-up duration^58^, and the number of BCTs identified in each RCT. Both subgroup analysis and meta-regression were also used to investigate potential sources of substantial heterogeneity when I^2^ ≥ 50%^49^.

### Supplementary information

Supplementary Information

## Data Availability

Y.Y.Y. has full access to all of the data in the study and takes responsibility for the integrity of the data and the accuracy of the data analysis. All study materials are available from the corresponding author upon reasonable request.

## Code availability

The code used for the analyses in R Studio (version 2022.07.1, ‘metafor’ package) is available upon reasonable request from the corresponding author.

## References

[1] World Health Organization. *Guideline: Sodium Intake for Adults and Children*. (World Health Organization, 2012). 23658998

[2] Mozaffarian D (2014). Global sodium consumption and death from cardiovascular causes. N. Engl. J. Med..

[3] World Health Organization. *Salt Reduction* https://www.who.int/news-room/fact-sheets/detail/salt-reduction (2020).

[4] Aliasgharzadeh S, Tabrizi JS, Nikniaz L, Ebrahimi-Mameghani M, Lotfi Yagin N (2022). Effect of salt reduction interventions in lowering blood pressure: a comprehensive systematic review and meta-analysis of controlled clinical trials. PLoS ONE.

[5] He FJ, Li J, MacGregor GA (2013). Effect of longer term modest salt reduction on blood pressure: cochrane systematic review and meta-analysis of randomised trials. Br. Med. J..

[6] Bibbins-Domingo K (2010). Projected effect of dietary salt reductions on future cardiovascular disease. N. Engl. J. Med..

[7] World Health Organization. *The SHAKE Technical Package for Salt Reduction*. 60 (World Health Organization, 2016).

[8] Trieu K (2017). Review of behaviour change interventions to reduce population salt intake. Int. J. Behav. Nutr. Phys. Act..

[9] Khalesi S (2022). Reducing salt intake: a systematic review and meta-analysis of behavior change interventions in adults. Nutr. Rev..

[10] O’Brien OA, McCarthy M, Gibney ER, McAuliffe FM (2014). Technology-supported dietary and lifestyle interventions in healthy pregnant women: a systematic review. Eur. J. Clin. Nutr..

[11] Ali SH (2019). Application of mobile health technologies aimed at salt reduction: systematic review. JMIR mHealth uHealth.

[12] He FJ (2022). App based education programme to reduce salt intake (AppSalt) in schoolchildren and their families in China: parallel, cluster randomised controlled trial. Br. Med. J..

[13] Payne Riches S (2021). A mobile health salt reduction intervention for people with hypertension: results of a feasibility randomized controlled trial. JMIR Mhealth Uhealth.

[14] Ashton LM (2019). Effectiveness of interventions and behaviour change techniques for improving dietary intake in young adults: a systematic review and meta-analysis of RCTs. Nutrients.

[15] Zandstra EH, Lion R, Newson RS (2016). Salt reduction: moving from consumer awareness to action. Food Qual. Prefer..

[16] Michie S (2013). The behavior change technique taxonomy (v1) of 93 hierarchically clustered techniques: building an international consensus for the reporting of behavior change interventions. Ann. Behav. Med.

[17] Carey RN (2019). Behavior change techniques and their mechanisms of action: a synthesis of links described in published intervention literature. Ann. Behav. Med..

[18] Cornelio ME (2014). Effect of a behavioral intervention of the SALdável program to reduce salt intake among hypertensive women: a randomized controlled pilot study. Eur. J. Cardiovasc. Nurs..

[19] Dorsch MP (2020). Effects of a novel contextual just-in-time mobile app intervention (LowSalt4Life) on sodium intake in adults with hypertension: pilot randomized controlled trial. JMIR Mhealth Uhealth.

[20] Dunbar SB (2005). Family education and support interventions in heart failure: a pilot study. Nurs. Res..

[21] Eyles H (2017). A salt-reduction smartphone app supports lower-salt food purchases for people with cardiovascular disease: Findings from the SaltSwitch randomised controlled trial. Eur. J. Prevent. Cardiol..

[22] Hwang JH (2014). Effects of intensive low-salt diet education on albuminuria among nondiabetic patients with hypertension treated with olmesartan: a single-blinded randomized, controlled trial. Clin. J. Am. Soc. Nephrol..

[23] Ipjian ML, Johnston CS (2017). Smartphone technology facilitates dietary change in healthy adults. Nutrition.

[24] Jarrar AH (2022). Using digital platform approach to reduce salt intake in a sample of UAE population: an intervention study. Front. Public Health.

[25] Morikawa N, Yamasue K, Tochikubo O, Mizushima S (2011). Effect of salt reduction intervention program using an electronic salt sensor and cellular phone on blood pressure among hypertensive workers. Clin. Exp. Hypertens..

[26] Nakadate M (2018). Effect of monitoring salt concentration of home-prepared dishes and using low-sodium seasonings on sodium intake reduction. Eur. J. Clin. Nutr..

[27] Takada T (2018). Effects of self-monitoring of daily salt intake estimated by a simple electrical device for salt reduction: a cluster randomized trial article. Hypertens. Res..

[28] Thatthong N (2020). Innovative tool for health promotion for at-risk Thai people with hypertension. J. Public Health.

[29] Toft U (2020). The effects of two intervention strategies to reduce the intake of salt and the sodium-to-potassium ratio on cardiovascular risk factors. a 4-month randomised controlled study among healthy families. Nutrients.

[30] Wiriyatanakorn S, Mukdadilok A, Kantachuvesiri S, Mekhora C, Yingchoncharoen T (2021). Impact of self-monitoring of salt intake by salt meter in hypertensive patients: a randomized controlled trial (SMAL-SALT). J. Clin. Hypertens..

[31] Yokokawa H (2020). An impact of dietary intervention on blood pressures among diabetic and/or hypertensive patients with high cardiovascular disorders risk in northern Thailand by cluster randomized trial. J. Gen. Fam. Med..

[32] Yuan YT, Zhou YF, Wang LM, Gong F (2019). Effect of mobile health education on salt restriction intervention in patients with salt sensitive hypertension (In Chinese). Chin. J. Soc. Med..

[33] Eyles H (2023). Effectiveness of a sodium-reduction smartphone app and reduced-sodium salt to lower sodium intake in adults with hypertension: findings from the salt alternatives randomized controlled trial. JMIR Mhealth Uhealth.

[34] Tan M, He F, Morris JK, MacGregor G (2022). Reducing daily salt intake in China by 1 g could prevent almost 9 million cardiovascular events by 2030: a modelling study. BMJ Nutr. Prev. Health.

[35] Filippini T (2021). Blood pressure effects of sodium reduction: dose-response meta-analysis of experimental studies. Circulation.

[36] Silva-Santos T (2021). Interventions that successfully reduced adults salt intake—a systematic review. Nutrients.

[37] Liu M (2022). A town-level comprehensive intervention study to reduce salt intake in China: cluster randomized controlled trial. Nutrients.

[38] Tam HL, Wong EML, Cheung K, Chung SF (2021). Effectiveness of text messaging interventions on blood pressure control among patients with hypertension: systematic review of randomized controlled trials. JMIR Mhealth Uhealth.

[39] Iribarren SJ (2017). Scoping review and evaluation of SMS/text messaging platforms for mHealth projects or clinical interventions. Int. J. Med. Inform..

[40] Brown IJ, Tzoulaki I, Candeias V, Elliott P (2009). Salt intakes around the world: implications for public health. Int. J. Epidemiol..

[41] Newson RS (2013). Barriers for progress in salt reduction in the general population. An international study. Appetite.

[42] Cheung J, Neyle D, Chow PPK (2021). Current knowledge and behavior towards salt reduction among Hong Kong citizens: a cross-sectional survey. Int. J. Environ. Res. Public Health.

[43] Prestwich A (2014). How can self-efficacy be increased? Meta-analysis of dietary interventions. Health Psychol. Rev..

[44] Page MJ (2021). The PRISMA 2020 statement: an updated guideline for reporting systematic reviews. Syst. Rev..

[45] Group KDIGOBPW (2021). KDIGO 2021 clinical practice guideline for the management of blood pressure in chronic kidney disease. Kidney Int..

[46] Byrd-Bredbenner C (2017). Systematic review of control groups in nutrition education intervention research. Int. J. Behav. Nutr. Phys. Act.

[47] World Health Organization. *How to obtain measures of population-level sodium intake in 24-hour urine samples*. (World Health Organization. Regional Office for the Eastern Mediterranean, 2018).

[48] Bakas T (2014). Evidence for stroke family caregiver and dyad interventions: a statement for healthcare professionals from the American Heart Association and American Stroke Association. Stroke.

[49] Higgins, J. P. et al. *Cochrane Handbook for Systematic Reviews of interventions*. (John Wiley & Sons, 2019).

[50] Sterne JAC (2019). RoB 2: a revised tool for assessing risk of bias in randomised trials. Br. Med. J..

[51] Lakens D (2013). Calculating and reporting effect sizes to facilitate cumulative science: a practical primer for t-tests and ANOVAs. Front. Psychol..

[52] Andrade C (2020). Mean difference, standardized mean difference (SMD), and their use in meta-analysis: as simple as it gets. J. Clin. Psychiatry.

[53] Cohen, J. *Statistical Power Analysis for the Behavioral Sciences*. (Routledge, 2013).

[54] Lin L (2018). Empirical comparison of publication bias tests in meta-analysis. J. Gen. Intern. Med..

[55] Duval S, Tweedie R (2000). Trim and fill: a simple funnel‐plot–based method of testing and adjusting for publication bias in meta‐analysis. Biometrics.

[56] Chan A, Chan SW-C, Khanam M, Kinsman L (2022). Factors affecting reductions in dietary salt consumption in people of Chinese descent: an integrative review. J. Adv. Nurs..

[57] Brandt CJ, Søgaard GI, Clemensen J, Søndergaard J, Nielsen JB (2018). Determinants of successful eHealth coaching for consumer lifestyle changes: qualitative interview study among health care professionals. J. Med. Internet Res..

[58] Huang L (2020). Effect of dose and duration of reduction in dietary sodium on blood pressure levels: systematic review and meta-analysis of randomised trials. Br. Med. J..

