# Supplementary material for: Technology-supported behavior change interventions for reducing sodium intake in adults: a systematic review and meta-analysis
Source: NPJ Digit Med. 2024 Mar 18;7:72. doi: 10.1038/s41746-024-01067-y (PMC10948864; doi:10.1038/s41746-024-01067-y)
Supplement: Supplementary file 1 — Supplementary Information [file 41746_2024_1067_MOESM1_ESM.pdf]

Supplementary Information

Contents

Supplementary Figure 1: Map Chart for the Study Implementation Country 2

Supplementary Figure 2: Risk of Bias Assessment 3

Supplementary Table 1: Additional Study Characteristics 4

Supplementary Table 2.1: The Behavior Change Technique Taxonomy (v1, BCTTv1) .. 9

Supplementary Table 2.2: BCTs Identified in Each Study 11

Supplementary Table 2.3: Frequency of BCTs 15

Supplementary Table 3: Subgroup Analysis of 24HUNa 17

Supplementary Table 4: Meta-Regression of 24HUNa 23

Supplementary Table 5: PRISMA 2020 Checklist 24

Supplementary Table 6: Search Query 28

Supplemental References 31

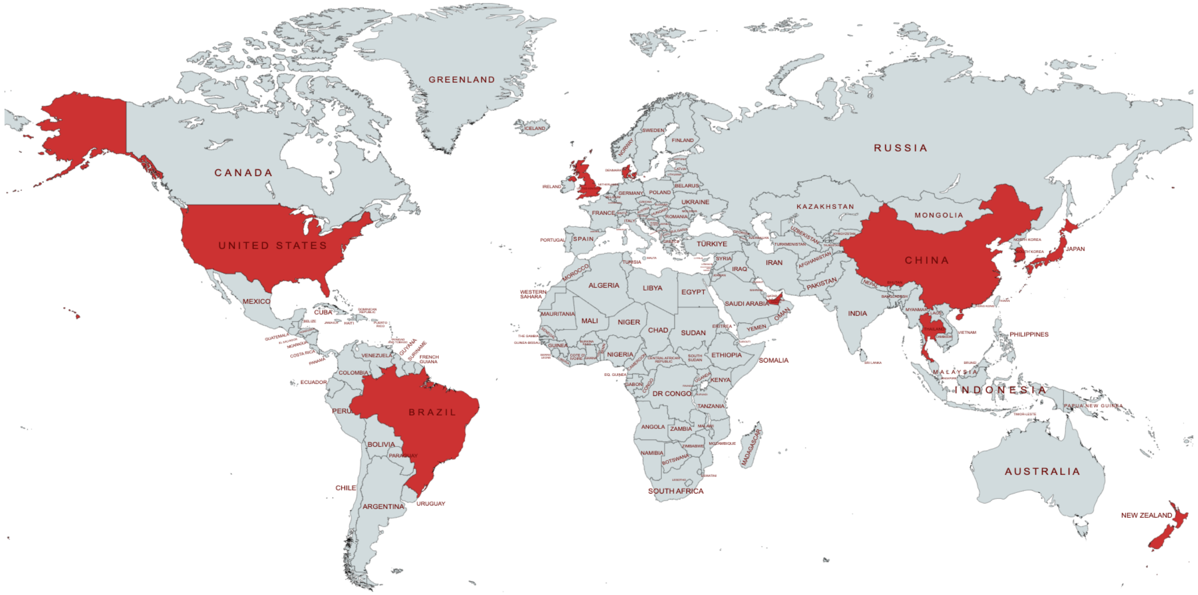

Supplementary Figure 1: Map Chart for the Study Implementation Country

Study ID	D1	D2	D3	D4	D5	Overall
Comelio 2014	!	-	+	!	!	-
Dorsch 2020	!	-	+	!	!	!
Dunbar 2005	!	-	+	!	!	!
Eyles a 2017	+	+	+	+	+	+
Eyles b 2023	+	+	+	+	+	+
He 2022	+	+	+	+	+	+
Hwang 2014	!	!	+	!	!	!
Ipjian 2017	+	+	+	+	+	!
Jarrar 2022	!	-	+	!	!	!
Morikawa 2011	!	+	+	+	+	!
Nakadate 2018	+	+	+	+	+	!
Riches 2021	+	+	+	+	+	+
Takada 2018	+	+	+	+	+	!
Thatthong 2020	!	-	-	+	!	-
Toft 2020	+	+	+	+	+	+
Wiriyatanakorn 2021	+	+	+	+	+	+
Yokokawa 2020	+	+	+	+	+	!
Yuan 2019	!	-	+	+	!	-

+	Low risk
!	Some concerns
-	High risk

D1	Randomisation process
D2	Deviations from the intended interventions
D3	Missing outcome data
D4	Measurement of the outcome
D5	Selection of the reported result

Supplementary Figure 2: Risk of Bias Assessment

Supplementary Table 1: Additional Study Characteristics

Author, Year, Country	Intervention Delivery Mode (No. of Families Involved)	Frequency	Delivery Professional	Theoretical Framework	Combined with Face-to-face Interventions	Method of Self-Estimation of Sodium Intake	Urine Sample Type	IG's Sodium Reduction Value (Proportion)	CG's Intervention
Cornelio[1], 2014, Brazil	Group-based (0)	Biweekly	Nurse + Nutritionist	TTM + SCT	Yes	Salt-restriction spoon	24-h urine (single urine sample)	-0.64 gm/24 h (-14.95%)	Inactive control: conventional treatment with general information about medication adherence, nutrition, and physical activity
Dorsch[2], 2020, USA	Individual-based (0)	When food shopping	NA	TPB + SRT + MDM	NA	App developed for sodium reduction	24-h urine (single urine sample)	-0.64 gm/24 h (-17.66%)	Inactive control: usual dietary advice
Dunbar[3], 2005, USA	Dyad-based (1)	NA	Nurse + Dietitian	SDT	Yes	NA	24-h urine (single urine sample)	-0.83 gm/24 h (-24.03%)	Active control: general heart failure information and sodium reduction education

Eyles a[4], 2017, New Zealand	Individual- or dyad-based (NA)	When food shopping	NA	NA	No	App developed for sodium reduction	Spot urine (single urine sample)	-0.09 gm/24 h (-2.51%)	Inactive control: usual care cardiac rehabilitation services
Eyles b[5], 2023, New Zealand	Individual-based (0)	When food shopping	NA	NA	No	App developed for sodium reduction	Spot urine (single urine sample)	+0.32 gm/24 h (+8.85%)	Active control: reduced-sodium salt and generic heart-healthy eating information
He[6], 2022, China	Group-based (2)	Monthly	Trained teachers	NA	Yes	App developed for sodium reduction	24-h urine (multiple urine samples: 2 samples were collected at each time point measurement)	-0.48 gm/24 h (-11.71%)	Inactive control: no treatment
Hwang[7], 2014, South Korea	Individual-based (0)	Weekly	Dietitian	NA	No	NA	24-h urine (single urine sample)	-0.74 gm/24 h (-20.91%)	Active control: routine low-salt diet education

Ipjian[8], 2017, USA	Individual-based (0)	Daily	NA	NA	NA	App developed for sodium reduction	Spot urine (multiple urine samples: 2 samples were collected at each time point measurement)	-0.84 gm/24 h (-20.03%)	Active control: paper tallying of estimated sodium intake
Jarrar[9], 2022, United Arab Emirates	Individual-based (0)	IG1: Weekly IG2: Biweekly	NA	NA	No	NA	24-h urine (single urine sample)	IG1: -0.28 gm/24 h (-9.21%) IG2: -0.17 gm/24 h (-5.46%)	Inactive control: no treatment
Morikawa[10], 2011, Japan	Individual-based (0)	NA	NA	NA	No	Digital self-monitoring sodium device	Spot urine (single urine sample)	-0.28 gm/24 h (NA)	Inactive control: no treatment
Nakadate[11], 2018, Japan	Individual- + dyad- + group-based (NA)	At least once a week	NA	NA	NA	Digital self-monitoring sodium device	24-h urine (single urine sample)	-0.78 gm/24 h (-21.23%)	Active control: general education on salt reduction and/or regular sodium seasoning

Riches[12], 2021, UK	Individual-based (0)	When food shopping	Nurse or healthcare assistant	BCW + TDF	Yes	App developed for sodium reduction	24-h urine (single urine sample)	-0.08 gm/24 h (-4.62%)	Active control: generic salt reduction advice leaflet
Takada[13], 2018, Japan	Dyad-based (1)	NA	Physician + Dietitian	SMT	NA	Digital self-monitoring sodium device	Spot urine (single urine sample)	-0.31 gm/24 h (-8.22%)	Active control: salt reduction lectures
Thatthong[14], 2020, Thailand	Individual-based (0)	Twice a week	Research assistant	NA	NA	NA	24-h urine (single urine sample)	NA	Inactive control: a general healthcare counseling program
Toft[15], 2020, Denmark	Dyad-based (1)	NA	Nutritionist	SCT	Yes	NA	24-h urine (multiple urine samples: 3 samples were collected at each time point measurement)	-0.70 gm/24 h (-18.92%)	Inactive control: bread with regular salt content
Wiriyatanakorn[16], 2021, Thailand	Group-based (0)	At least five times per week	Dietitian	NA	Yes	Digital self-monitorin	24-h urine (single urine sample)	-0.73 gm/24 h (-20.74%)	Active control: dietary education

						g sodium device			(with sodium reduction)
Yokokawa[17], 2020, Thailand	Group-based (0)	NA	Dietitian	NA	Yes	Digital self-monitoring sodium device	Spot urine (single urine sample)	-0.66 gm/24 h (-9.23%)	Inactive control: routine care services and a brief individual health education session (not focused on salt reduction)
Yuan[18], 2019, China	Individual-based (0)	Weekly	Nurse	NA	Yes	Scale	24-h urine (single urine sample)	-2.77 gm/24 h (-46.94%)	Inactive control: routine outpatient follow-up

Abbreviation: App: application; BCW: behavior change wheel; CG: control group; gm/24 h: gram/ 24 hours; IG: intervention group; MDM: mindful decision making; NA: not available; No.: number; SCT: social cognitive theory; SDT: self-determination theory; SMS: short message service; SMT: self-monitoring theory; SRT: self-regulation theory; TDF: theoretical domains framework; TPB: theory of planned behavior; TTM: the transtheoretical model; UK: the United Kingdom; USA: the United States of America.

Note:

1. Intervention delivery mode was defined as individual-based interventions (delivered to one participant), dyad-based interventions (delivered to two participants), or group-based interventions (delivered to three or more participants).
2. Proportion of sodium reduction in the intervention groups was calculated as the change in 24-h urinary sodium from post-intervention to baseline divided by the 24-h urinary sodium at baseline. The formula was: 100%* (24HUNa at post-intervention - 24HUNa at baseline)/24HUNa at baseline.

Supplementary Table 2.1: The Behavior Change Technique Taxonomy (v1, BCTTv1)

Grouping and BCTs[19]	Grouping and BCTs	Grouping and BCTs
**1. Goals and planning**	**6. Comparison of behavior**	**12. Antecedents**
1.1. Goal setting (behavior)	6.1. Demonstration of the behavior	12.1. Restructuring the physical environment
1.2. Problem solving	6.2. Social comparison	12.2. Restructuring the social environment
1.3. Goal setting (outcome)	6.3. Information about others' approval	12.3. Avoidance/reducing exposure to cues for the behavior
1.4. Action planning	**7. Associations**	12.4. Distraction
1.5. Review behavior goal(s)	7.1. Prompts/cues	12.5. Adding objects to the environment
1.6. Discrepancy between current behavior and goal	7.2. Cue signaling reward	12.6. Body changes
1.7. Review outcome goal(s)	7.3. Reduce prompts/cues	**13. Identity**
1.8. Behavioral contract	7.4. Remove access to the reward	13.1. Identification of self as role model
1.9. Commitment	7.5. Remove aversive stimulus	13.2. Framing/reframing
**2. Feedback and monitoring**	7.6. Satiation	13.3. Incompatible beliefs
2.1. Monitoring of behavior by others without feedback	7.7. Exposure	13.4. Valued self-identify
2.2. Feedback on behavior	7.8. Associative learning	13.5. Identity associated with changed behavior
2.3. Self-monitoring of behavior	**8. Repetition and substitution**	**14. Scheduled consequences**
2.4. Self-monitoring of outcome(s) of behavior	8.1. Behavioral practice/rehearsal	14.1. Behavior cost
2.5. Monitoring of outcome(s) of behavior without feedback	8.2. Behavior substitution	14.2. Punishment
2.6. Biofeedback	8.3. Habit formation	14.3. Remove reward
2.7. Feedback on outcome(s) of behavior	8.4. Habit reversal	14.4. Reward approximation
**3. Social support**	8.5. Overcorrection	14.5. Rewarding completion
3.1. Social support (unspecified)	8.6. Generalisation of target behavior	14.6. Situation-specific reward
3.2. Social support (practical)	8.7. Graded tasks	14.7. Reward incompatible behavior
3.3. Social support (emotional)	**9. Comparison of outcomes**	14.8. Reward alternative behavior
**4. Shaping knowledge**	9.1. Credible source	14.9. Reduce reward frequency
4.1. Instruction on how to	9.2. Pros and cons	14.10. Remove punishment
	9.3. Comparative imagining of future outcomes	**15. Self-belief**

perform the behavior<br>4.2. Information about Antecedents<br>4.3. Re-attribution<br>4.4. Behavioral experiments		15.1. Verbal persuasion about capability<br>15.2. Mental rehearsal of successful performance<br>15.3. Focus on past success<br>15.4. Self-talk
**5. Natural consequences**	**10. Reward and threat**	**16. Covert learning**
5.1. Information about health consequences<br>5.2. Salience of consequences<br>5.3. Information about social and environmental consequences<br>5.4. Monitoring of emotional consequences<br>5.5. Anticipated regret<br>5.6. Information about emotional consequences	10.1. Material incentive (behavior)<br>10.2. Material reward (behavior)<br>10.3. Non-specific reward<br>10.4. Social reward<br>10.5. Social incentive<br>10.6. Non-specific incentive<br>10.7. Self-incentive<br>10.8. Incentive (outcome)<br>10.9. Self-reward<br>10.10. Reward (outcome)<br>10.11. Future punishment	16.1. Imaginary punishment<br>16.2. Imaginary reward<br>16.3. Vicarious consequences
	**11. Regulation**	
	11.1. Pharmacological support<br>11.2. Reduce negative emotions<br>11.3. Conserving mental resources<br>11.4. Paradoxical instructions	

Supplementary Table 2.2: BCTs Identified in Each Study

BCT Taxonomy Code	Cornelio et al.[1]	Dorsch et al.[2]	Dunbar et al.[3]	Eyles a et al.[4]	Eyles b et al.[5]	He et al.[6]	Hwang et al. [7]	Ipjian et al.[8]	Jarrar et al. [9]
1.1. Goal setting (behavior)	+	+		+	+	+		+	
1.2. Problem solving	+		+			+			
1.3. Goal setting (outcome)	+	+	+			+	+	+	
1.4. Action planning	+	+	+	+	+	+			
1.5. Review behavior goal(s)						+			
1.6. Discrepancy between current behavior and goal						+			
1.7. Review outcome goal(s)						+			
2.2. Feedback on behavior	+	+	+	+	+		+		
2.3. Self-monitoring of behavior						+			
2.4. Self-monitoring of outcome(s) of behavior		+						+	
2.6. Biofeedback			+						
2.7. Feedback on outcome(s) of behavior		+	+			+		+	
3.2. Social support (practical)	+		+			+			
3.3. Social support (emotional)			+						
4.1. Instruction on how to perform the behavior	+	+	+	+	+	+	+	+	+
4.4. Behavioral experiments									
5.1. Information about health consequences	+	+	+			+			+
5.3. Information about social and environmental consequences									
6.1. Demonstration of the behavior	+	+	+	+	+	+	+	+	+
6.2. Social comparison						+			
7.1. Prompts/cues		+		+	+	+			+
8.1. Behavioral practice/rehearsal	+	+	+	+	+	+	+	+	+
8.2. Behavior substitution	+	+	+	+	+	+			+

8.3. Habit formation	+	+	+	+	+	+		+	+
8.4. Habit reversal	+	+	+	+	+	+			+
9.1. Credible source	+	+	+						
10.2. Material reward (behavior)						+			
10.4. Social reward						+			
11.3. Conserving mental resources									
12.1. Restructuring the physical environment						+			
12.2. Restructuring the social environment						+			
12.5. Adding objects to the environment						+			
13.1. Identification of self as role model									
13.2. Framing/reframing	+								
15.1. Verbal persuasion about capability									
**Total No. of BCT**	**15**	**15**	**16**	**10**	**10**	**24**	**5**	**8**	**8**

Supplementary Table 2.2 (Continued)

**BCT Taxonomy Code**	**Morikawa et al.**[10]	**Nakadate et al.**[11]	**Riches et al.**[12]	**Takada et al.**[13]	**Thatthong et al.**[14]	**Toft et al.**[15]	**Wiriyatanakorn et al.**[16]	**Yokokawa et al.**[17]	**Yuan et al.**[18]
1.1. Goal setting (behavior)		+	+	+		+	+		+
1.2. Problem solving			+			+			+
1.3. Goal setting (outcome)								+	+
1.4. Action planning		+	+	+		+	+		+
1.5. Review behavior goal(s)						+			+
1.6. Discrepancy between current behavior and goal						+			
1.7. Review outcome goal(s)						+			+
2.2. Feedback on behavior		+	+			+			+
2.3. Self-monitoring of behavior		+	+				+		+

2.4. Self-monitoring of outcome(s) of behavior	+		+	+			+	+	
2.6. Biofeedback	+			+				+	+
2.7. Feedback on outcome(s) of behavior	+		+	+				+	+
3.2. Social support (practical)						+	+		+
3.3. Social support (emotional)									
4.1. Instruction on how to perform the behavior	+	+	+	+	+	+	+	+	+
4.4. Behavioral experiments								+	
5.1. Information about health consequences			+	+		+		+	+
5.3. Information about social and environmental consequences		+							
6.1. Demonstration of the behavior	+	+		+	+	+	+	+	+
6.2. Social comparison									
7.1. Prompts/cues	+		+		+	+			
8.1. Behavioral practice/rehearsal	+	+		+	+	+	+	+	+
8.2. Behavior substitution			+					+	
8.3. Habit formation	+	+		+	+	+	+		+
8.4. Habit reversal									
9.1. Credible source			+	+		+	+	+	+
10.2. Material reward (behavior)									
10.4. Social reward									+
11.3. Conserving mental resources			+						
12.1. Restructuring the physical environment									
12.2. Restructuring the social environment									

12.5. Adding objects to the environment			+						
13.1. Identification of self as role model			+						
13.2. Framing/reframing									
15.1. Verbal persuasion about capability			+						
**Total No. of BCT**	**8**	**9**	**16**	**11**	**5**	**15**	**10**	**11**	**18**

Note: The grid with "+" means the BCT was identified in that RCT's intervention.
Abbreviation: BCT: behavior change technique; No.: number.

Supplementary Table 2.3: Frequency of BCTs

BCT Taxonomy Code	No. of Study Used the BCT	Percentage of Study Used the BCT (%)
4.1 Instruction on how to perform the behavior	18	100.00%
6.1 Demonstration of the behavior	17	94.44%
8.1 Behavioral practice/rehearsal	17	94.44%
8.3 Habit formation	15	83.33%
1.1 Goal setting (behavior)	12	66.67%
1.4 Action planning	12	66.67%
2.2 Feedback on behavior	10	55.56%
5.1 Information about health consequences	10	55.56%
2.7 Feedback on outcome(s) of behavior	9	50.00%
7.1 Prompts/cues	9	50.00%
8.2 Behavior substitution	9	50.00%
9.1 Credible source	9	50.00%
1.3 Goal setting (outcome)	8	44.44%
2.4 Self-monitoring of outcome(s) of behavior	7	38.89%
8.4 Habit reversal	7	38.89%
1.2 Problem solving	6	33.33%
3.2 Social support (practical)	6	33.33%
2.3 Self-monitoring of behavior	5	27.78%
2.6 Biofeedback	5	27.78%
1.5 Review behavior goal(s)	3	16.67%
1.7 Review outcome goal(s)	3	16.67%
1.6 Discrepancy between current behavior and goal	2	11.11%
10.4 Social reward	2	11.11%
12.5 Adding objects to the environment	2	11.11%
3.3 Social support (emotional)	1	5.56%
4.4 Behavioral experiments	1	5.56%
5.3 Information about social and environmental consequences	1	5.56%
6.2 Social comparison	1	5.56%

10.2 Material reward (behavior)	1	5.56%
11.3 Conserving mental resources	1	5.56%
12.1 Restructuring the physical environment	1	5.56%
12.2 Restructuring the social environment	1	5.56%
13.1 Identification of self as role model	1	5.56%
13.2 Framing/reframing	1	5.56%
15.1Verbal persuasion about capability	1	5.56%

Abbreviation: BCTs: behavior change techniques; No.: number.

Supplementary Table 3: Subgroup Analysis of 24HUNa

Subgroups	No. of Study	IG's Sample Size	CG's Sample Size	MD (95% CI)	SMD (95% CI)	P Value of Subgroup Difference
**Blood Pressure of Participants**						
Hypertensive	7	449	438	-0.36 (-0.69, -0.02)	-0.23 (-0.45, -0.01)	P=0.86
Normative	3	160	78	-0.42 (-0.65, -0.18)	-0.57 (-0.85, -0.29)	
Hypertensive + Normative	4	1075	1070	-0.34 (-0.45, -0.23)	-0.26 (-0.36, -0.16)	
**Primary Technology Type**						
Instant messaging App	2	125	103	-0.62 (-1.17, -0.07)	-0.53 (-0.80, -0.26)	P=0.49
Telephone call	4	276	236	-0.51 (-0.78, -0.24)	-0.33 (-0.51, -0.16)	
App developed for sodium reduction	6	781	764	-0.27 (-0.51, -0.02)	-0.21 (-0.45, -0.04)	
Digital self-monitoring sodium device	4	526	525	-0.30 (-0.51, -0.09)	-0.37 (-0.60, -0.13)	
**Intervention Delivery Professional**						
Multidisciplinary professionals	3	173	165	-0.30 (-0.46, -0.13)	-0.35 (-0.61, -0.09)	P=0.17
Unidisciplinary professional	7	1310	1289	-0.48 (-0.67, -0.28)	-0.31 (-0.43, -0.18)	
**Intervention Delivery Mode**						
Individual-based	7	439	390	-0.37 (-0.64, -0.10)	-0.30 (-0.53, -0.07)	P=0.97
Dyad-based*	3	174	132	-0.36 (-0.60, -0.13)	-0.50 (-0.73, -0.26)	
Group-based†	4	1075	1071	-0.39	-0.24	

				(-0.53, -0.26)	(-0.34, -0.13)	
**Family Members Involved**						
Yes	4	768	722	-0.34<br>(-0.46, -0.23)	-0.33<br>(-0.46, -0.19)	P=0.61
No	10	920	871	-0.40<br>(-0.60, -0.21)	-0.28<br>(-0.44, -0.12)	
**Partially or Entirely Technology-Supported**						
Partially (combined with face-to-face component)	8	1285	1224	-0.46<br>(-0.67, -0.25)	-0.29<br>(-0.41, -0.16)	P=0.30
Entirely	8	464	424	-0.33<br>(-0.45, -0.21)	-0.37<br>(-0.55, -0.18)	
**Intervention Frequency**						
≤Weekly (i.e., more frequent)	6	332	311	-0.61<br>(-0.82, -0.40)	-0.48<br>(-0.64, -0.32)	P=0.03
>Weekly (i.e., less frequent)	3	697	667	-0.34<br>(-0.48, -0.20)	-0.25<br>(-0.36, -0.14)	
**Intervention Setting**						
Nonhealthcare setting	10	1104	1016	-0.36<br>(-0.47, -0.26)	-0.30<br>(-0.43, -0.17)	P=0.41
Healthcare setting	3	451	453	-0.56<br>(-0.83, -0.29)	-0.34<br>(-0.62, -0.07)	
Nonhealthcare + healthcare setting	3	194	179	-0.33<br>(-0.88, 0.22)	-0.33<br>(-0.70, 0.03)	
**Method of Self-Estimation of Sodium Intake**						
App developed for sodium reduction	6	781	764	-0.27<br>(-0.51, -0.02)	-0.21<br>(-0.45, 0.04)	P=0.70
Digital self-monitoring sodium device	4	526	525	-0.30<br>(-0.51, -0.09)	-0.37<br>(-0.60, -0.13)	
Salt-restriction spoon/scale	2	146	141	-0.60<br>(-1.33, 0.13)	-0.32<br>(-0.72, 0.08)	
**Urine Sample Used for Estimating Sodium Intake**						

24-h urine (single + multiple urine samples)	12	2510	2405	-0.38 (-0.58, -0.18)	-0.30 (-0.46, -0.14)	P=1.00
24-h urine (single urine sample)	10	1853	1791	-0.38 (-0.63, -0.12)	-0.30 (-0.50, -0.10)	
24-h urine (multiple urine samples)	2	657	614	-0.38 (-0.53, -0.22)	-0.29 (-0.49, -0.10)	
Spot urine	5	585	590	-0.24 (-0.41, -0.07)	-0.30 (-0.56, -0.04)	
**Control Group**						
Active control	8	433	416	-0.41 (-0.69, -0.12]	-0.33 (-0.56, -0.10)	P=0.70
Inactive control	8	1316	1232	-0.38 (-0.49, -0.27)	-0.28 (-0.42, -0.21)	
**BCT: 1.1 Goal Setting (Behavior)**						
Yes	12	1142	1075	-0.38 (-0.55, -0.22)	-0.32 (-0.47, -0.17)	P=0.79
No	4	607	573	-0.41 (-0.58, -0.24)	-0.27 (-0.40, -0.15)	
**BCT: 1.2 Problem Solving**						
Yes	5	866	800	-0.43 (-0.73, -0.12)	-0.29 (-0.47, -0.12)	P=0.74
No	10	799	764	-0.37 (-0.48, -0.26)	-0.37 (-0.49, -0.24)	
**BCT: 1.3 Goal Setting (Outcome)**						
Yes	8	1304	1306	-0.46 (-0.61, -0.31)	-0.27 (-0.37, -0.18)	P=0.23
No	7	361	258	-0.33 (-0.48, -0.19)	-0.46 (-0.63, -0.30)	
**BCT: 1.4 Action Planning**						
Yes	12	1159	1089	-0.37 (-0.53, -0.22)	-0.31 (-0.45, -0.17)	P=0.66

No	4	590	559	-0.42 (-0.59, -0.25)	-0.33 (-0.52, -0.15)	
**BCT: 2.2 Feedback on Behavior**						
Yes	10	560	501	-0.39 (-0.63, -0.15)	-0.28 (-0.46, -0.11)	P=0.80
No	6	1189	1147	-0.36 (-0.46, -0.26)	-0.34 (-0.47, -0.20)	
**BCT: 2.3 Self-monitoring of Behavior**						
Yes	5	782	757	-0.50 (-0.88, -0.11)	-0.34 (-0.55, -0.13)	P=0.50
No	10	883	807	-0.36 (-0.47, -0.25)	-0.33 (-0.45, -0.22)	
**BCT: 2.4 Self-monitoring of Outcome(s) of Behavior**						
Yes	6	568	560	-0.39 (-0.65, -0.12)	-0.32 (-0.56, -0.09)	P=0.94
No	9	1097	1004	-0.40 (-0.51, -0.29)	-0.32 (-0.41, -0.24)	
**BCT: 2.6 Biofeedback**						
Yes	4	569	571	-0.50 (-0.79, -0.21)	-0.37 (-0.58, -0.16)	P=0.46
No	11	1096	993	-0.38 (-0.49, -0.27)	-0.33 (-0.45, -0.22)	
**BCT: 2.7 Feedback on Outcome(s) of Behavior**						
Yes	8	1233	1218	-0.41 (-0.60, -0.22)	-0.30 (-0.44, -0.17)	P=0.99
No	7	432	346	-0.41 (-0.55, -0.26)	-0.41 (-0.55, -0.26)	
**BCT: 3.2 Social Support (Practical)**						
Yes	6	880	829	-0.53 (-0.76, -0.30)	-0.35 (-0.49, -0.21)	P=0.14
No	9	785	735	-0.34	-0.34	

				(-0.46, -0.23)	(-0.49, -0.20)	
**BCT: 5.1 Information about Health Consequences**						
Yes	10	1425	1323	-0.35 (-0.47, -0.24)	-0.29 (-0.40, -0.19)	P=0.19
No	5	240	241	-0.52 (-0.75, -0.30)	-0.47 (-0.65, -0.28)	
**BCT: 6.1 Demonstration of the Behavior**						
Yes	15	1718	1632	-0.39 (-0.49, -0.29)	-0.33 (-0.43, -0.23)	P=0.04
No	1	31	16	0.32 (-0.36, 1.00)	0.27 (-0.34, 0.87)	
**BCT: 7.1 Prompts/Cues**						
Yes	6	827	728	-0.33 (-0.45, -0.22)	-0.32 (-0.48, -0.15)	P=0.10
No	9	838	836	-0.53 (-0.72, -0.33)	-0.36 (-0.50, -0.23)	
**BCT: 8.1 Behavioral Practice/Rehearsal**						
Yes	15	1718	1632	-0.39 (-0.49, -0.29)	-0.33 (-0.43, -0.23)	P=0.04
No	1	31	16	0.32 (-0.36, 1.00)	0.27 (-0.34, 0.87)	
**BCT: 8.2 Behavior Substitution**						
Yes	9	1316	1253	-0.33 (-0.43, -0.22)	-0.21 (-0.33, -0.09)	P=0.05
No	7	433	395	-0.60 (-0.85, -0.35)	-0.48 (-0.62, -0.34)	
**BCT: 8.3 Habit Formation**						
Yes	13	1225	1127	-0.39 (-0.50, -0.27)	-0.36 (-0.49, -0.24)	P=0.72
No	3	524	521	-0.31 (-0.70, 0.08)	-0.20 (-0.41, 0.01)	

**BCT: 8.4 Habit Reversal**						
Yes	7	911	858	-0.33 (-0.45, -0.22)	-0.25 (-0.41, -0.10)	P=0.17
No	9	838	790	-0.50 (-0.73, -0.28)	-0.38 (-0.54, -0.22)	
**BCT: 9.1 Credible Source**						
Yes	9	794	739	-0.43 (-0.65, -0.22)	-0.33 (-0.48, -0.17)	P=0.66
No	6	871	825	-0.38 (-0.49, -0.27)	-0.31 (-0.41, -0.22)	

Abbreviation: App: application; BCT: behavior change technique; CG: control group; CI: confidence interval; IG: intervention group; MD: mean difference; NA: not available; No.: number; SMD: standardized mean difference; 24HUNa: 24-h urinary sodium.

[*]: The dyad-based intervention mode refers to the intervention delivered to 2 people; †: The group-based intervention mode refers to the intervention delivered to ≥ 3 people.

Supplementary Table 4: Meta-Regression of 24HUNa

**Intervention Characteristics**	**Coefficient (95% CI)**	**SE**	**P Value**
Sample Size	0.00 (-0.00, 0.00)	0.00	1.00
Proportion of Female Participants	0.15 (-0.43, 0.72)	0.29	0.62
Mean Age	0.00 (-0.01, 0.01)	0.00	0.71
Intervention Duration	-0.00 (-0.02, 0.02)	0.01	0.76
Follow-up Duration	-0.01 (-0.05, 0.03)	0.02	0.66
Number of BCTs Identified in Each Study	0.00 (-0.01, 0.01)	0.01	0.93

Abbreviation: BCT: behavior change technique; CI: confidence interval; SE: standard error; 24HUNa: 24-h urinary sodium.

Supplementary Table 5: PRISMA 2020 Checklist

Section and Topic[20]	Item #	Checklist item	Location where item is reported
**TITLE**			
Title	1	Identify the report as a systematic review.	Title Page
**ABSTRACT**			
Abstract	2	See the PRISMA 2020 for Abstracts checklist.	P2
**INTRODUCTION**			
Rationale	3	Describe the rationale for the review in the context of existing knowledge.	P4-5
Objectives	4	Provide an explicit statement of the objective(s) or question(s) the review addresses.	P5
**METHODS**			
Eligibility criteria	5	Specify the inclusion and exclusion criteria for the review and how studies were grouped for the syntheses.	P15
Information sources	6	Specify all databases, registers, websites, organizations, reference lists and other sources searched or consulted to identify studies. Specify the date when each source was last searched or consulted.	P14
Search strategy	7	Present the full search strategies for all databases, registers and websites, including any filters and limits used.	Supplementary Table 5
Selection process	8	Specify the methods used to decide whether a study met the inclusion criteria of the review, including how many reviewers screened each record and each report retrieved, whether they worked independently, and if applicable, details of automation tools used in the process.	P15-16
Data collection process	9	Specify the methods used to collect data from reports, including how many reviewers collected data from each report, whether they worked independently, any processes for obtaining or confirming data from study investigators, and if applicable, details of automation tools used in the process.	P15-16

Section and Topic[20]	Item #	Checklist item	Location where item is reported
Data items	10a	List and define all outcomes for which data were sought. Specify whether all results that were compatible with each outcome domain in each study were sought (e.g. for all measures, time points, analyses), and if not, the methods used to decide which results to collect.	P15-16
	10b	List and define all other variables for which data were sought (e.g. participant and intervention characteristics, funding sources). Describe any assumptions made about any missing or unclear information.	P15-16
Study risk of bias assessment	11	Specify the methods used to assess risk of bias in the included studies, including details of the tool(s) used, how many reviewers assessed each study and whether they worked independently, and if applicable, details of automation tools used in the process.	P17
Effect measures	12	Specify for each outcome the effect measure(s) (e.g. risk ratio, mean difference) used in the synthesis or presentation of results.	P17
Synthesis methods	13a	Describe the processes used to decide which studies were eligible for each synthesis (e.g. tabulating the study intervention characteristics and comparing against the planned groups for each synthesis (item #5)).	P16
	13b	Describe any methods required to prepare the data for presentation or synthesis, such as handling of missing summary statistics, or data conversions.	P16
	13c	Describe any methods used to tabulate or visually display results of individual studies and syntheses.	Table 1, Figure 2-4
	13d	Describe any methods used to synthesize results and provide a rationale for the choice(s). If meta-analysis was performed, describe the model(s), method(s) to identify the presence and extent of statistical heterogeneity, and software package(s) used.	P17
	13e	Describe any methods used to explore possible causes of heterogeneity among study results (e.g. subgroup analysis, meta-regression).	P17

Section and Topic[20]	Item #	Checklist item	Location where item is reported
	13f	Describe any sensitivity analyses conducted to assess robustness of the synthesized results.	P17
Reporting bias assessment	14	Describe any methods used to assess risk of bias due to missing results in a synthesis (arising from reporting biases).	P17
Certainty assessment	15	Describe any methods used to assess certainty (or confidence) in the body of evidence for an outcome.	P17
**RESULTS**			
Study selection	16a	Describe the results of the search and selection process, from the number of records identified in the search to the number of studies included in the review, ideally using a flow diagram.	Figure 1
	16b	Cite studies that might appear to meet the inclusion criteria, but which were excluded, and explain why they were excluded.	Not Applicable
Study characteristics	17	Cite each included study and present its characteristics.	Table 1
Risk of bias in studies	18	Present assessments of risk of bias for each included study.	Supplementary Figure 2
Results of individual studies	19	For all outcomes, present, for each study: (a) summary statistics for each group (where appropriate) and (b) an effect estimate and its precision (e.g. confidence/credible interval), ideally using structured tables or plots.	Figure 2-4
Results of syntheses	20a	For each synthesis, briefly summarise the characteristics and risk of bias among contributing studies.	P7-8, Figure 2-4
	20b	Present results of all statistical syntheses conducted. If meta-analysis was done, present for each the summary estimate and its precision (e.g. confidence/credible interval) and measures of statistical heterogeneity. If comparing groups, describe the direction of the effect.	P7-8, Figure 2-4
	20c	Present results of all investigations of possible causes of heterogeneity among study results.	P8-9, Supplementary Table 2-3
	20d	Present results of all sensitivity analyses conducted to assess the robustness	P8-9

Section and Topic[20]	Item #	Checklist item	Location where item is reported
		of the synthesized results.	
Reporting biases	21	Present assessments of risk of bias due to missing results (arising from reporting biases) for each synthesis assessed.	P7-8
Certainty of evidence	22	Present assessments of certainty (or confidence) in the body of evidence for each outcome assessed.	P7-8
**DISCUSSION**			
Discussion	23a	Provide a general interpretation of the results in the context of other evidence.	P9-13
	23b	Discuss any limitations of the evidence included in the review.	P14
	23c	Discuss any limitations of the review processes used.	Not Applicable
	23d	Discuss implications of the results for practice, policy, and future research.	P9-13
**OTHER INFORMATION**			
Registration and protocol	24a	Provide registration information for the review, including register name and registration number, or state that the review was not registered.	P14
	24b	Indicate where the review protocol can be accessed, or state that a protocol was not prepared.	P14
	24c	Describe and explain any amendments to information provided at registration or in the protocol.	Not Applicable
Support	25	Describe sources of financial or non-financial support for the review, and the role of the funders or sponsors in the review.	P35
Competing interests	26	Declare any competing interests of review authors.	P35
Availability of data, code and other materials	27	Report which of the following are publicly available and where they can be found: template data collection forms; data extracted from included studies; data used for all analyses; analytic code; any other materials used in the review.	P19

Supplementary Table 6: Search Query

Search Query in PubMed (URL: https://pubmed.ncbi.nlm.nih.gov/)

#	**Query**
#1	(internet-based intervention(MeSH Terms)) OR (internet based intervention)
#2	((((((telemedicine(MeSH Terms)) OR (mobile health)) OR (mHealth)) OR (electronic health)) OR (eHealth)) OR (digital health)) OR (telehealth)
#3	(((text messaging(MeSH Terms)) OR (short message service*)) OR (SMS)) OR (instant message)
#4	((((((((((((((((((((mobile applications(MeSH Terms)) OR (APP)) OR (software)) OR (wearable electronic devices(MeSH Terms))) OR (wireless device)) OR (blue-tooth)) OR (sensor)) OR (cell phone(MeSH Terms))) OR (phone)) OR (email)) OR (video)) OR (game)) OR (interactive voice response)) OR (visual reality)) OR (technology*)) OR (web-based)) OR (online)) OR (internet)) OR (computer)) OR (laptop)) OR (tablet)) OR (social media)) OR (artificial intelligence)
#5	#1 OR #2 OR #3 OR #4
#6	(((sodium, dietary(MeSH Terms)) OR (sodium chloride(MeSH Terms))) OR (salt)) OR (sodium)
#7	(((((((consum*) OR (intake)) OR (eat*)) OR (diet*)) OR (food)) OR (nutrition*)) OR (meal*)) OR (dietetics)
#8	((((((((((((((((((((reduc*) OR (restrict*)) OR (promot*)) OR (limit*)) OR (minimi*)) OR (low*)) OR (free)) OR (health education(MeSH Terms))) OR (educat*)) OR (manag*)) OR (excret*)) OR (substitute*)) OR (composition)) OR (risk reduction behaviour(MeSH Terms))) OR (behaviour therapy(MeSH Terms))) OR (healthy lifestyle(MeSH Terms))) OR (lifestyle)) OR (lifestyle intervention)) OR (activit*)) OR (project)) OR (campaign)) OR (initiative)
#9	#6 AND #7 AND #8
#10	(animals(MeSH Terms)) NOT (humans(MeSH Terms))
#11	#5 AND #9 NOT #10
#12	#5 AND #9 NOT #10, Fliter: Chinese, English, from 2000/1/1 – 2023/4/13

Search Query in Cochrane Library (URL: https://www.cochranelibrary.com/)

#	**Query**
#1	internet-based intervention or information communication technology or telemedicine or mobile health or mHealth or electronic health or eHealth or digital health or telehealth or text messaging or short message service or SMS or instant message or mobile applications

	or APP or software or wearable electronic devices or wireless device or blue tooth or sensor or cell phone or phone or email or video or game or interactive voice response or visual reality or technology or web-based or online or internet or computer or laptop or tablet or social media or artificial intelligence
#2	sodium or sodium chloride or salt
#3	consum* or intake or eat* or diet* or food or nutrition* or meal* or dietetics
#4	reduc* or restrict* or promot* or limit* or minimi* or low* or free or health education or educat* or manag* or excret* or substitute* or composition or ingest* or risk reduction behaviour or behaviour therapy or healthy lifestyle or lifestyle or lifestyle intervention or activit* or project or campaign or initiative
#5	#2 AND #3 AND #4
#6	#1 AND #5<br>with Publication Year from 2000 to 2023, with Cochrane Library publication date from Jan 2000 to Apr 2023, in Trials

Search Query in Embase (URL: https://www.elsevier.com/solutions/embase-biomedical-research)

#	Query
#1	(internet-based intervention or information communication technology or telemedicine or mobile health or mHealth or electronic health or eHealth or digital health or telehealth or text messaging or short message service or SMS or instant message or mobile applications or APP or software or wearable electronic devices or wireless device or blue tooth or sensor or cell phone or phone or email or video or game or interactive voice response or visual reality or technology or web-based or online or internet or computer or laptop or tablet or social media or artificial intelligence).mp.
#2	(sodium or sodium chloride or salt).mp.
#3	(consum* or intake or eat* or diet* or food or nutrition* or meal* or dietetics).mp.
#4	(reduc* or restrict* or promot* or limit * or minimi* or low* or free or health education or educat* or manag* or excret* or substitute* or composition or ingest* or risk reduction behaviour or behaviour therapy or healthy lifestyle or lifestyle or lifestyle intervention or activit* or project or campaign or initiative).mp.
#5	#2 and #3 and #4
#6	#1 and #5
#7	limit #6 to (human and (English language or Chinese) and yr="2000 - Current")

Search Query in Wan Fang (URL: http://www.wanfangdata.com/)

#	Filters	Query
#1	摘要	盐 AND (摄入 OR 消耗 OR 食用 OR 使用 OR 代谢 OR 管理 OR 教育 OR 代替 OR 替代 OR 低钠)

#2	摘要	钠 AND (摄入 OR 消耗 OR 食用 OR 使用 OR 代谢 OR 管理 OR 教育 OR 代替 OR 替代)
#3	摘要	“减盐”OR “限盐” OR “控盐” OR “少盐” OR “低盐” OR “降盐”
#4	摘要	手机 OR 平板电脑 OR 电脑 OR 软件 OR 应用程序 OR 移动应用 OR 微信 OR “APP” OR 可穿戴设备
#5	摘要	互联网 OR “远程医疗”OR “数字医疗” OR “数字健康” OR “移动医疗” OR “移动健康” OR 大数据 OR 人工智能
#6		(#1 OR #2 OR #3) AND (#4 OR #5) with “time limit: 2000-2023” and “ 医药卫生” and “期刊论文”

Search Query in China National Knowledge Infrastructure (URL: https://chn.oversea.cnki.net/index/)

#	Filters	Query
#1	篇关摘	((盐 * (摄入 + 消耗 + 食用 + 使用 + 代谢 + 管理 + 教育 + 代替 + 替代 + 低钠) + (钠 * (摄入+ 消耗 + 食用 + 使用 + 代谢 + 管理 + 教育 + 代替 + 替代))
#2	篇关摘	“减盐” + “限盐” + “控盐” + “少盐” + “低盐” + “降盐”
#3	篇关摘	手机 + 平板电脑 + 电脑 + 软件 + 应用程序 + 移动应用 + 微信 + “APP” + 可穿戴设备 + 互联网 + “远程医疗” + “数字医疗” + “数字健康” + “移动医疗” + “移动健康” + 大数据 + 人工智能
#4		(#1 OR #2) AND #3 with “time limit: 01/01/2000-13/04/2023”

Supplemental References

1 Cornelio, M. E. *et al.* Effect of a behavioral intervention of the SALdável program to reduce salt intake among hypertensive women: a randomized controlled pilot study. *European Journal of Cardiovascular Nursing* **15**, e85-e94 (2014). https://doi.org:10.1177/1474515115589275

2 Dorsch, M. P. *et al.* Effects of a novel contextual just-in-time mobile app intervention (LowSalt4Life) on sodium intake in adults with hypertension: pilot randomized controlled trial. *JMIR Mhealth Uhealth* **8**, e16696 (2020). https://doi.org:10.2196/16696

3 Dunbar, S. B. *et al.* Family education and support interventions in heart failure: a pilot study. *Nursing Research* **54**, 158-166 (2005). https://doi.org:10.1097/00006199-200505000-00003

4 Eyles, H. *et al.* A salt-reduction smartphone app supports lower-salt food purchases for people with cardiovascular disease: Findings from the SaltSwitch randomised controlled trial. *European Journal of Preventive Cardiology* **24**, 1435-1444 (2017). https://doi.org:10.1177/2047487317715713

5 Eyles, H. *et al.* Effectiveness of a sodium-reduction smartphone app and reduced-sodium salt to lower sodium intake in adults with hypertension: findings from the salt alternatives randomized controlled trial. *JMIR Mhealth Uhealth* **11**, e43675 (2023). https://doi.org:10.2196/43675

6 He, F. J. *et al.* App based education programme to reduce salt intake (AppSalt) in schoolchildren and their families in China: parallel, cluster randomised controlled trial. *BMJ* **376 (no pagination)**, e066982 (2022). https://doi.org:10.1136/bmj-2021-066982

7 Hwang, J. H. *et al.* Effects of intensive low-salt diet education on albuminuria among nondiabetic patients with hypertension treated with olmesartan: a single-blinded randomized, controlled trial. *Clinical Journal of the American Society of Nephrology* **9**, 2059-2069 (2014). https://doi.org:10.2215/CJN.01310214

8 Ipjian, M. L. & Johnston, C. S. Smartphone technology facilitates dietary change in healthy adults. *Nutrition* **33**, 343-347 (2017). https://doi.org:10.1016/j.nut.2016.08.003

9 Jarrar, A. H. *et al.* Using digital platform approach to reduce salt intake in a sample of UAE population: an intervention study. *Frontiers in Public Health* **10**, 860835 (2022). https://doi.org:10.3389/fpubh.2022.860835

10 Morikawa, N., Yamasue, K., Tochikubo, O. & Mizushima, S. Effect of salt reduction intervention program using an electronic salt sensor and cellular phone on blood pressure among hypertensive workers. *Clinical and Experimental Hypertension* **33(4)**, 216-222 (2011). https://doi.org:10.3109/10641963.2011.583966

11 Nakadate, M. *et al.* Effect of monitoring salt concentration of home-prepared dishes and using low-sodium seasonings on sodium intake reduction. *European Journal of Clinical Nutrition* **72**, 1413-1420 (2018). https://doi.org:10.1038/s41430-017-0053-2

12 Payne Riches, S. *et al.* A mobile health salt reduction intervention for people with hypertension: results of a feasibility randomized controlled trial. *JMIR Mhealth Uhealth* **9**, e26233 (2021). https://doi.org:10.2196/26233

13 Takada, T. *et al.* Effects of self-monitoring of daily salt intake estimated by a simple electrical device for salt reduction: a cluster randomized trial article. *Hypertension Research* **41**, 524-530 (2018). https://doi.org:10.1038/s41440-018-0046-0
14 Thatthong, N. *et al.* Innovative tool for health promotion for at-risk Thai people with hypertension. *Journal of Public Health (Germany)* **28**, 437-443 (2020). https://doi.org:10.1007/s10389-019-01028-w
15 Toft, U. *et al.* The effects of two intervention strategies to reduce the intake of salt and the sodium-to-potassium ratio on cardiovascular risk factors. a 4-month randomised controlled study among healthy families. *Nutrients* **12**, 1467 (2020). https://doi.org:10.3390/nu12051467
16 Wiriyatanakorn, S., Mukdadilok, A., Kantachuvesiri, S., Mekhora, C. & Yingchoncharoen, T. Impact of self-monitoring of salt intake by salt meter in hypertensive patients: a randomized controlled trial (SMAL-SALT). *Journal of Clinical Hypertension (Greenwich, Conn.)* **23**, 1852-1861 (2021). https://doi.org:10.1111/jch.14344
17 Yokokawa, H. *et al.* An impact of dietary intervention on blood pressures among diabetic and/or hypertensive patients with high cardiovascular disorders risk in northern Thailand by cluster randomized trial. *J Gen Fam Med* **22**, 28-37 (2020). https://doi.org:10.1002/jgf2.379
18 Yuan, Y. T., Zhou, Y. F., Wang, L. M. & Gong, F. Effect of mobile health education on salt restriction intervention in patients with salt sensitive hypertension (In Chinese). *Chinese Journal of Social Medicine* **36**, 281-284 (2019). https://doi.org:10.3969/j.issn.1673-5625.2019.03.017
19 Michie, S. *et al.* The behavior change technique taxonomy (v1) of 93 hierarchically clustered techniques: building an international consensus for the reporting of behavior change interventions. *Annals of Behavioral Medicine* **46**, 81-95 (2013). https://doi.org:10.1007/s12160-013-9486-6
20 Page, M. J. *et al.* The PRISMA 2020 statement: an updated guideline for reporting systematic reviews. *Systematic Reviews* **10**, 1-11 (2021). https://doi.org:10.1186/s13643-021-01626-4
